# The mitochondrial genome of *Chelipoda* sp. (Diptera: Empididae)

**DOI:** 10.1080/23802359.2020.1768945

**Published:** 2020-05-27

**Authors:** Shang Gao, Yue Liu, Jiale Zhou, Ding Yang

**Affiliations:** College of Plant Protection, China Agricultural University, Beijing, China

**Keywords:** Mitochondrial genome, Hemerodromiinae, phylogenetics

## Abstract

The dance fly *Chelipoda* sp. belongs to the subfamily Hemerodromiinae of Empididae. The mitogenome (GenBank accession number: MT396991) of *Chelipoda* sp. was sequenced, the first representative of the mitogenome of the subfamily. The nearly complete mitogenome is 14,976 bp totally, consisting of 13 protein-coding genes, 2 rRNAs, and 22 transfer RNAs. All genes have the similar locations and strands compared with that of other published species of Empididae. The nucleotide composition biases toward A and T, which together made up 77.2％of the entirety. Bayesian inference analysis strongly supported the monophyly of Empidoidea, Empididae and Dolichopodidae. The phylogenetic relationship within Empidoidea is as follows: (Dolichopodinae + Neurigoninae) + (((Empidinae + Hemerodromiinae) + (Trichopezinae + Oreogetoninae)) + Ocydromiinae) in this study.

Empididae is one of the largest families in Diptera with over 5000 described species from the world. The adults and larvae of Empididae are predatory. They are the natural enemies of crop pests, fruit tree pests, tree pests and health pests, and play a certain role in controlling pests. This group has large number of individuals, wide appetite, strong predatory ability, and is a useful natural enemy insect resource (Yang et al. 2007).

The specimens of *Chelipoda* sp. used for this study were collected in Zhouzhi County of Shaanxi by Xuankun Li and identified by Ding Yang. Specimens are deposited in the Entomological Museum of China Agricultural University (CAU) with the accession number CAUYD3012 (Room 2005, Plant Protection Building, West Campus, China Agricultural University). The total genomic DNA was extracted from the whole body (except head) of the specimen using the QIAamp DNA Blood Mini Kit (Qiagen, Germany) and stored at –20 °C until needed. The mitogenome was sequenced in BaiNuoDaCheng Biotechnology Company used NGS. The nearly complete mitogenome of *Chelipoda* sp. is 14,976 bp (GenBank accession number: MT396991). It encoded 13 PCGs, 22 tRNA genes, and 2 rRNA genes and were similar with the related reports published before (Yang et al. 2019; Hou et al. 2019; Qilemoge et al. 2019, 2020). All genes have the locations and strands similar to other published Empididae species. The nucleotide composition of the mitogenome was biased toward A and T, with 77.2% of A + T content (A = 38.9%, T = 38.3%, C = 13.5%, G = 9.2%). The A + T content of PCGs, tRNAs, and rRNAs is 75.6%, 79.6%, and 82.4%, respectively. The total length of all 13 PCGs of *Chelipoda* sp. is 11,261 bp. Five PCGs (*NAD2*, *ATP8, NAD3, NAD5, NAD6*) initiated with ATT codons, and six PCGs (*COII*, *COIII*, *ATP6*, *NAD4*, *NAD4L* and *CYTB*) initiated with ATG codons, and *COI* and *NAD1* initiated with CCG and TTG as a start codon, respectively. Eleven PCGs used the typical termination codons TAA except *NAD5* used TAG and *NAD4* used TA in *Chelipoda* sp.

Phylogenetic analysis was performed based on the nucleotide sequences of 13 PCGs from 10 Diptera species. Bayesian (BI) analysis generated the phylogenetic tree topologies based on the PCGs matrices (Figure 1). The phylogenetic result shows that the monophyly of Empidoidea, Dolichopodidae and Empididae were strongly supported. The monophyletic Dolichopodidae that contains Dolichopodinae and Neurigoninae was assigned to the sister group to the clade of Empididae that consists of Empidinae, Hemerodromiinae, Trichopezinae, Oreogetoninae, Ocydromiinae in this study. It is clear that the phylogenetic relationship within Empidoidea is as follows: (Dolichopodinae + Neurigoninae) + (((Empidinae + Hemerodromiinae) + (Trichopezinae + Oreogetoninae)) + Ocydromiinae) in this study. This result shows that Empidinae is the sister group to Hemerodromiinae, which is consistent with the phylogenetic result of the previous research (Yang et al. 2007). The mitogenome of *Chelipoda* sp. could provide the important information for the further studies of Empidoidea phylogeny.

**Figure 1.**
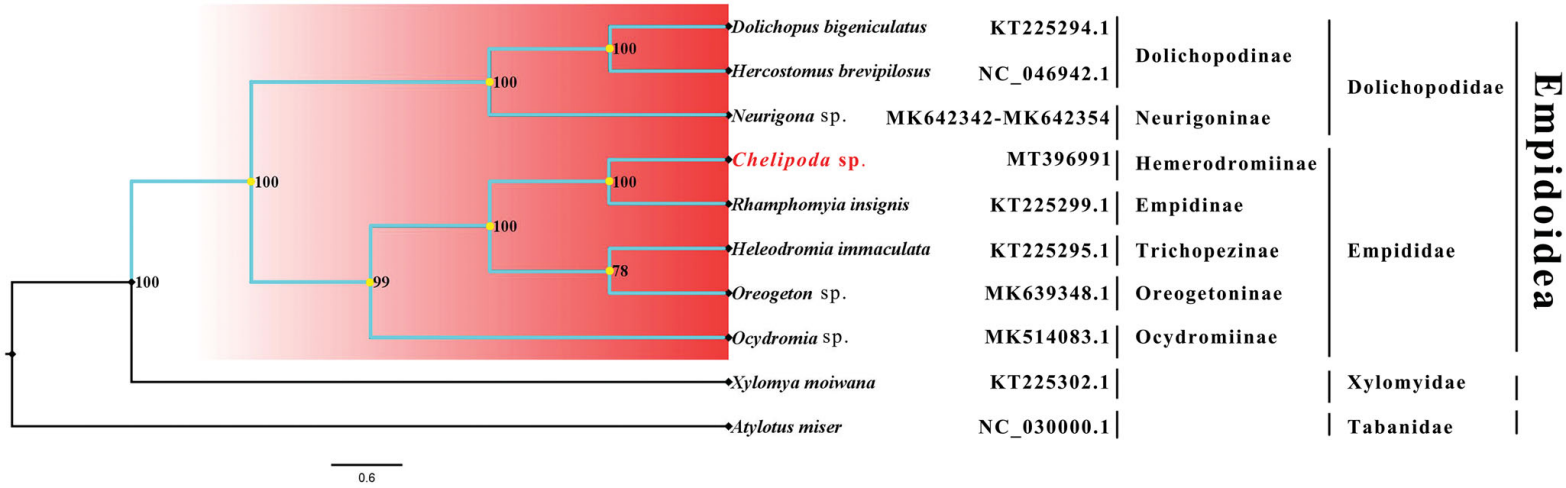
Bayesian phylogenetic tree of 10 Diptera species. The posterior probabilities are labeled at each node. Genbank accession numbers of all sequence used in the phylogenetic tree have been included in the figure and corresponding to the names of all species.

## Data Availability

The data that support the findings of this study are openly available in [NCBI] at [https://www.ncbi.nlm.nih.gov/], reference number [MT396991].
